# Adapting harm reduction services during COVID-19: lessons from the supervised injecting facilities in Australia

**DOI:** 10.1186/s12954-021-00471-x

**Published:** 2021-02-17

**Authors:** Amanda Roxburgh, Marianne Jauncey, Carolyn Day, Mark Bartlett, Shelley Cogger, Paul Dietze, Suzanne Nielsen, Julie Latimer, Nico Clark

**Affiliations:** 1 Health Risks Program, Burnet Institute, Melbourne, Australia; 2 Discipline of Addiction Medicine, the Central Clinical School, Sydney Medical School, the Faculty of Medicine and Health, University of Sydney, Sydney, Australia; 3 National Drug and Alcohol Research Centre, UNSW, Sydney, Australia; 4 Monash Addiction Research Centre, Monash University, Melbourne, Australia; 5 Uniting Medically Supervised Injecting Centre, Sydney, Australia; 6 North Richmond Community Health Medically Supervised Injecting Room, Melbourne, Australia; 7 Addiction Medicine, Royal Melbourne Hospital, Melbourne, Australia; 8 Department of Pharmacology, University of Adelaide, Adelaide, Australia

**Keywords:** Harm reduction, Supervised injecting facilities, Drug consumption rooms, COVID-19, People who inject drugs

## Abstract

The COVID-19 crisis has had profound impacts on health service provision, particularly those providing client facing services. Supervised injecting facilities and drug consumption rooms across the world have been particularly challenged during the pandemic, as have their client group—people who consume drugs. Several services across Europe and North America closed due to difficulties complying with physical distancing requirements. In contrast, the two supervised injecting facilities in Australia (the Uniting Medically Supervised Injecting Centre—MSIC—in Sydney and the North Richmond Community Health Medically Supervised Injecting Room—MSIR—in Melbourne) remained open (as at the time of writing—December 2020). Both services have implemented a comprehensive range of strategies to continue providing safer injecting spaces as well as communicating crucial health information and facilitating access to ancillary services (such as accommodation) and drug treatment for their clients. This paper documents these strategies and the challenges both services are facing during the pandemic. Remaining open poses potential risks relating to COVID-19 transmission for both staff and clients. However, given the harms associated with closing these services, which include the potential loss of life from injecting in unsafe/unsupervised environments, the public and individual health benefits of remaining open are greater. Both services are deemed ‘essential health services’, and their continued operation has important benefits for people who inject drugs in Sydney and Melbourne.

## Introduction

Public health interventions in response to COVID-19, including border closures and physical distancing measures, have had profound implications for the ways in which people access health services [1]. Service providers have faced significant challenges to remain open during the pandemic, and many (e.g. general practitioners and mental health clinicians) have adapted by moving operations online [2]. This is not possible for many services where ongoing client facing operations are essential. This is particularly the case for supervised injecting facilities (SIFs) and drug consumption rooms (DCRs) more broadly.

Supervised injecting facilities are designed to provide highly marginalised and stigmatised communities who consume drugs [3] with; (1) safer spaces to inject drugs; (2) immediate responses to overdose and other adverse drug reactions (where necessary); and (3) facilitated access to a range of other health and social services. In reality, SIFs provide much more than these services (e.g. responding to acute mental health issues, harm reduction advice and community engagement), highlighting their flexibility and responsiveness in meeting the often complex health and social needs of their clients [4].

There are two SIFs currently operating in Australia, the Uniting Medically Supervised Injecting Centre (MSIC) in Sydney and the North Richmond Community Health Medically Supervised Injecting Room (MSIR) in Melbourne. Each service operates under specific legislation that allows exemption from criminal liability for both clients and staff, and with explicit support from state police. In New South Wales and Victoria, police are encouraged to exercise discretion not to charge people for simple drug possession if they are in the vicinity of, or travelling to and from, the service.

The MSIC is located in Kings Cross in Sydney, an area that has historically had large street-based illicit drug markets in operation [3]. Staff working at the MSIC include a medical director, operations manager, nurse unit manager, mental health nurse coordinator, referral coordinator, health education officers, registered nurses and security staff. The MSIC has 16 injecting spaces available for client use. It is a stand-alone service with close links to many other health, social and harm reduction services. It is an injecting facility and as such, does not cater for other modes of drug administration.

The MSIR is located in North Richmond in Melbourne, an area that has historically recorded high levels of public heroin use and related harms [5, 6]. Staff working at the MSIR include a medical director, operations manager, nurse team leader, registered nurses, harm reduction practitioners and security staff. The MSIR has 20 injecting spaces available. It is co-located with a community health centre with medical staff, dental staff, drug outreach workers and allied health workers. In addition, a consulting zone located within the injecting room building provides a range of on-site services provided by NRCH and partner organisations including BBV testing and treatment, opioid agonist treatment, basic oral health care (including with silver fluoride), housing and legal services, alcohol and drug treatments, mental health counselling). As an injecting facility the MSIR also only caters for this mode of drug administration.

Since opening in 2001, the MSIC in Sydney has supported over 1.1 million injecting visits and responded to over 10,000 overdoses (personal communication, M Bartlett, 11 August 2020). According to the MSIR Review Panel’s final report, in its first 18 months the Melbourne MSIR responded to 2657 overdoses [6]. These services are important not only in mitigating comorbidities associated with non-fatal overdose, and in reducing the number of injections that occur in public places, but also in averting fatal overdose, highlighted by the fact that neither facility has ever had a single fatality.

People who inject drugs are likely to be impacted by COVID-19 in unique ways and for multiple reasons. First, the introduction of stay-at-home and isolation orders presents risks for people injecting alone, as the presence of others is often a protective factor for overdose [7].

Second, a large proportion of people who attend SIFs are either street based, homeless or in boarding houses [8] and may have no home in which to self-isolate, increasing their risk of contracting the virus. While state governments across the country have made provisions to temporarily accommodate these communities during the pandemic [9], there may be increased risks in these contexts including overcrowding, difficulties maintaining physical distancing, and challenges with early detection and sufficient isolation of potential COVID-19 cases [10].

Third, many people who inject drugs have poor physical health and underlying respiratory conditions [11], which may increase susceptibility to poorer outcomes if they contract COVID-19. Finally, any disruptions to the illicit drug market may have a range of consequences, including people potentially reducing their use and seeking drug treatment, but also people potentially moving to more dangerous substances (e.g. fentanyl or fentanyl analogues) or heavier and more frequent patterns of use [12]. Indeed, in the context of increasing numbers of overdose deaths attributed to fentanyl recorded in the region during the pandemic, fentanyl has been described in North America as the ‘predictable outcome of opioid prohibition’ [13].

The challenges presented by the COVID-19 pandemic have impacted many harm reduction and treatment services worldwide [1]. Several DCRs across Europe have been forced to close, particularly where distancing was not possible, while others adapted by providing outdoor services [14]. Many drop-in centres across Europe providing support for people who consume drugs have also been closed [14]. Harm reduction services have also closed or restricted intake in North America [13, 15, 16], as have some Australian alcohol and other drug (AOD) treatment services, creating further risks for people who inject drugs, and stress on local health services [17].

As at the time of writing, the two SIFs operating in Australia have remained open throughout the pandemic by adopting a range of adaptive practice changes. Lessons learnt in responding to the pandemic are likely to apply to a range of other client facing services including homelessness services, outreach needle and syringe programmes (NSP), and opioid agonist treatment (OAT) [1].

This paper documents the strategies implemented by the MSIC and MSIR in adapting to COVID-19 between March and December 2020. It does so in the context of the implementation of government restrictions in Australia nationally and in New South Wales and Victoria. It also discusses future challenges that may arise for clients of these services as the pandemic evolves.

### Government restrictions in Australia, February to December 2020

**Fig. 1:**
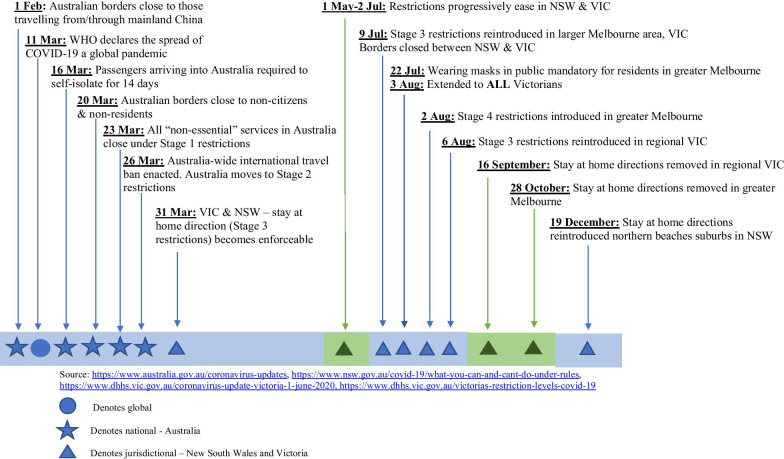
Timeline of relevant government restrictions in Australia, New South Wales (NSW) and Victoria (VIC), February–December 2020. *Source*: https://www.australia.gov.au/coronavirus-updates, https://www.nsw.gov.au/covid-19/what-you-can-and-cant-do-under-rules, https://www.dhhs.vic.gov.au/coronavirus-update-victoria-1-june-2020, https://www.dhhs.vic.gov.au/victorias-restriction-levels-covid-19

Figure 1 outlines some of the major Australian jurisdictional responses to the COVID-19 pandemic up to the date of writing (December 2020), documenting the dates of border closure and restrictions on gatherings and movement. Legislation governing these restrictions occurs at the jurisdictional and national level, and there is variation in how they are implemented. Victoria and New South Wales are the most populated jurisdictions in Australia [18] and account for the greatest number of COVID-19 cases during the pandemic so far [19].

Australian borders were closed to non-residents and non-citizens from 20 March. More restrictive lockdown measures were progressively introduced throughout March, culminating in enforceable stay-at-home directions for all Australians from 31 March (Stage 3 restrictions).

The process of easing restrictions commenced on 1 May in New South Wales [20], and from 11 May in Victoria.

On 9 July, Stage 3 restrictions were reintroduced for Victorian residents living in greater Melbourne and the Mitchell Shire, following several large clusters of COVID-19 cases occurring in the state. Stage 4 restrictions, including a night-time curfew (8 pm–5 am), were introduced in greater Melbourne on 3 August [21]. New South Wales also recorded several localised outbreaks in early July, and again in December, with stay at home restrictions reintroduced for parts of the greater Sydney area on 19 December (Fig. 1).

### Cases reported in New South Wales and Victoria

**Fig. 2:**
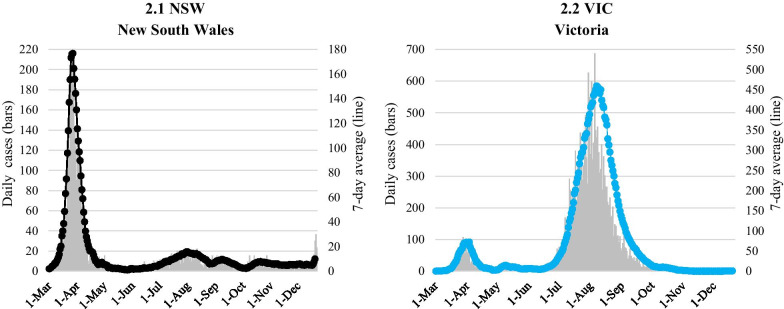
All confirmed COVID-19 cases: 7-day moving average in NSW and VIC, March—December 2020. *Source*: www.covid19data.com.au accessed 15 January 2021

Figure 2a, b shows the number of cases and seven day moving average reported in New South Wales and Victoria. New South Wales experienced a large spike in daily cases in late March (212 cases), while Victoria experienced a second and much larger spike (723 cases) in late July [19]. For the period 1 March to 31 December 2020, New South Wales had a cumulative total of 4,731 cases, equating to 58.2 cases per 100,000 population, and Victoria had a cumulative total of 20,358 cases equating to 306.1 cases per 100,000 population.

### Procedural changes

The MSIC and MSIR implemented a range of strategies from as early as 16 March aimed at reducing the COVID risk to staff and clients (Table 1), with many responses being escalated as necessary throughout this period.

**Table 1:** Procedural changes: Uniting Medically Supervised Injecting Centre (MSIC) and North Richmond Community Health Medically Supervised Injecting Room (MSIR)

MSIC	MSIR
*Screening—clients and staff*	
Screening (including travel history, symptoms and temperature) of all clients entering the service. Symptomatic clients were assessed and referred for testing. All MSIC staff similarly screened	Screening (including travel history, symptoms and temperature) of all clients entering the service. Symptomatic clients tested onsite (asymptomatic clients tested periodically as per state directives). Installation of a mass fever screening system. All MSIR staff similarly screened
*Exclusion from the service—clients and staff*	
*Exclusion criteria:* Returned from international travel in preceding 14 days (MSIC and MSIR); considered a close contact because visited specified COVID-19 hotspots in preceding 14 days (MSIC only); any known exposure to the virus in the preceding 14 days (MSIC and MSIR); MSIR clients with unexplained fever, or fever and COVID-19 symptoms. From 22 July, MSIR clients not agreeing to wear a mask not permitted entry (masks provided onsite)	
*Physical distancing and other changes within the services—clients and staff*	
*Overall*—MSIC staff to remain 1.5 m distance from clients while they are using the service except in case of emergency. All client-facing staff to wear personal protective equipment (PPE) in clinical areas (as below). Hourly cleaning of clinical areas. Encouraging client time spent in the service to be less than one hour, unless they require monitoring due to decreased level of consciousness or post-overdose	*Overall*—MSIR staff to remain 1.5 m distance from clients while they are using the service except in case of emergency. All client-facing staff to wear PPE in clinical areas (as below). Hourly cleaning of clinical areas. Air conditioning settings changed and air purifiers with HEPA filters in all client areas. Implementation of a support role to guide clients through service and procedural changes. Encouraging clients to reduce time spent in the service and exit as soon as safe to do so
*Registration area*—Controlling entry through the door, reducing number of clients in this area to a maximum of three, and two staff to comply with capacity per square metre and physical distancing, barriers placed at the registration desk. MSIC staff to wear surgical masks and protective eyewear in this area	*Registration area*—Controlling entry through the door, reducing number of clients in this area to a maximum of six (four MSIR and two NSP/clinic clients) to comply with capacity per square metre and physical distancing. MSIR staff to wear N95 masks, face shields, gloves and gowns in this area
*Injecting area*—Making each booth single person only, with the option for two double booths and a maximum of 12 clients from 7 July. All MSIC staff to wear N95 masks and protective eyewear in this area	*Injecting area*—Closing every 2nd booth (reducing injecting positions from 20 to 13). Limiting vein care to less than 15 min. All MSIR staff to wear N95 masks, faces shields, gloves and gowns in this area
*Aftercare area*—Reduced seating available in this area. Refreshments removed. Promotion of opioid agonist treatment (OAT) referral options and take-home naloxone (THN) with training encouraged for all clients. Limiting time clients spend in aftercare area. Collection of feedback from clients on changes in service provision during COVID-19. All MSIC staff to wear surgical masks and protective eyewear in this area	*Aftercare area*—Reduced seating available in this area. Refreshments removed. Offering clients OAT, overdose/THN training and nicotine replacement therapy (NRT). Limiting time clients spend in aftercare area. All MSIR staff to wear N95 masks, face shields, gloves and gowns in this area
*Overdose response*	
All MSIC staff to wear full PPE—gown, face shield, N95 mask and gloves when responding to an overdose that may require bag valve mask (BVM) resuscitation. All MSIC staff responding to an overdose with oxygen only are required to wear N95 masks and protective eyewear—no gown or gloves are required for these responses	All MSIR staff to wear full PPE (gown, full face shield, N95 mask and gloves) when responding to an overdose. Implementation of a scribe at MSIR to record observations and support staff with communication
*MSIC* changed first response to overdose: ceased BVM resuscitation for clients who are apnoeic (not breathing) due to aerosolization risk. Replaced by immediate naloxone administration (below)	*MSIR* changed first response to overdose: oxygen flow limited to 6 l/min, use of non-rebreather masks if 6 l/min with a Hudson mask is not sufficient. No nebulising medication administered. Severe overdoses moved to (enclosed) medical monitoring room using evacuation slide sheet and crash mat for response, which may include ventilatory support
Naloxone (800 mcg IMI) administered immediately for apnoeic clients, in place of BVM—repeat in 2 to 3 min as necessary	Naloxone (1200 mcg IMI) administered immediately for apnoeic clients (800 mcg IMI if some breathing but less than 5 breaths per minute)
Commence BVM *only* if client not responsive to naloxone	Commence BVM in enclosed medical monitoring room using two-handed vice grip on the mask to reduce aerosolization risk
Reduction in oxygen flow to 6 l/min when administering oxygen through Hudson mask, due to aerosolization risk. Staff encouraged to turn oxygen off before removing Hudson mask to reduce any slight risk of aerosolization	Reduction in oxygen flow to 6 l/min using non-rebreather mask if required
*Tracking and testing—clients and staff*	
Facilitated access to COVID-19 swab testing	COVID-19 swab testing onsite
Tracking register implemented for staff onsite—record of risk exposure (e.g. responded to overdose) absences, COVID tests and results	
“Stage Log” for all MSIC staff, documenting time in and time out of each stage during working shift. Also in staff kitchen area where staff have breaks MSIC management team seating changed so that alternative teams placed in different ‘air space’ to reduce contact and transmission risk. Shift patterns of front-line staff unable to be altered due to smaller staff numbers	MSIR staff movements recorded through the service to assist with contact tracing, if necessary. Activation of the COVIDsafe app on two service phones to facilitate contract tracing MSIR has onsite CCTV (since inception) (not available at MSIC) MSIR staff divided into teams to further reduce contact between staff and limit number of contacts in the event of a confirmed case. Shift patterns altered to reduce staff crossover
	MSIR recommissioned a consulting room in the *Registration area* as a ‘single occupant/single use’ alternative injecting and aftercare area for clients who meet COVID-19 testing criteria and who agree to an onsite test. This is an additional mechanism to support COVID-19 testing, self-isolation and isolation planning, and reduce risks of transmission due to non-disclosure/masking of COVID-19 symptoms
*Education about virus transmission risks—clients and staff*	
At least weekly briefings with clinical staff about implementation of and changes to procedures in relation to COVID-19 and transmission risks. Informal audits done on donning and doffing of PPE and reminders about mask hygiene. Weekly teleconferences between the MSIC and MSIR to discuss ongoing response and procedural changes. Specific COVID-19 pandemic training undertaken by MSIR staff	
Educational material about the virus, including local harm reduction messages, about the importance of hand hygiene, cough etiquette, physical distancing and testing placed throughout all service and office areas. Conversations with clients occurring daily as part of service provision. Ongoing monitoring of adherence to COVID-19 government directives	
*Ancillary services support—clients*	
Facilitating access to temporary accommodation	Facilitating access to short-term accommodation through co-located onsite housing officer. Facilitating access to telehealth, including drug treatment services, income support and legal services
Promotion of referral options for OAT	Providing onsite OAT (i.e. long-acting injectable buprenorphine), and as part of a COVID-19 outreach service for clients in short-term accommodation
Mental health support provided by specialist onsite mental health clinicians	
Advocacy in relation to legal issues arising (e.g. policing of COVID-19 restrictions) through lockdown periods	

The MSIC and MSIR differ in terms of service location, and overall service structure. The MSIR is a programme of North Richmond Community Health (NRCH), a community health provider. NRCH and partner organisations provide co-located health and social support services in addition to the supervised injecting facility. In contrast, MSIC is a standalone supervised injecting facility

### Physical service changes to facilitate physical distancing

Procedures controlling entry to the services, including manning the front door and reducing the number of clients at one time in the registration area, were implemented across both services. Staff members/security staff at each service ensured clients were distancing appropriately, while they waited to access the service. The total number of injecting spaces was reduced (from 16 to 8 at the MSIC; from 20 to 13 at the MSIR), and clients were asked to limit the time they spent in the service. All client-facing staff at both services were required to wear personal protective equipment (PPE) in clinical service areas (Table 1).

### Screening, testing and contact tracing

Clients at both services were screened for travel history, symptoms and temperature prior to entry. MSIC clients with symptoms were offered a face mask and referred for testing at the nearest facility. Those who met COVID-19 testing criteria were not permitted to access the service. Local AOD clinical services offered pop-up and outreach COVID testing.

Testing procedures at the MSIR evolved in response to changing levels of community transmission. In the initial phases of the pandemic, clients were encouraged to undergo onsite COVID-19 testing. Clients who were symptomatic were not permitted entry to the service at this stage. In the context of increasing community transmission rates in Victoria, all MSIR clients who were symptomatic were required to undergo testing. During this time, expert advice from Infection Prevention and Control consultants was sought. Recommendations included the implementation of an enclosed alternative injecting and aftercare space for individual use for clients who met COVID-19 testing criteria. Deep cleaning procedures were implemented for this area after every occasion of use.

Staff of both services were also screened for temperature and movements prior to commencing work in each service.

Support for contact tracing in both services was critical to enable a prompt response in the event of a COVID-19 case being identified among staff or clients. This was facilitated through the online clinical databases at each service, with the clients’ time in and out of each stage, and the number of the injecting space recorded electronically. MSIR also used closed circuit television (CCTV) monitoring, which is a standard feature at the service. Staff monitoring was facilitated through documentation of staff movements through each stage (Table 1).

### Overdose response

Overdose response protocols were changed at both facilities including: (1) staff to wear full PPE when responding to overdose; (2) earlier administration of intramuscular naloxone; and (3) cessation of bag valve mask (BVM) resuscitation (unless absolutely necessary) and reduction in oxygen flow where oxygen-assisted respiration was required, due to the aerosolization risks associated with both procedures.

### Ancillary services

Facilitating access to temporary accommodation and OAT during the pandemic has been a substantial and important undertaking for both services. Provision of onsite OAT commenced at the MSIR in September 2019; however, clients were increasingly encouraged to commence treatment during the pandemic. MSIC also actively encouraged referrals to OAT. There has been increased uptake of OAT (particularly buprenorphine depot) during this time among MSIR and MSIC clients. The facilitation of access to short-term accommodation for clients dramatically increased across both services due to government initiatives implemented in Australia during the pandemic. Both services also increased the provision of take-home naloxone (THN) during this time.

### Challenges

Several of these procedural changes are unique to the SIF environment. Airway management during overdose response undoubtedly increases the risks of COVID-19 transmission. The aerosolization risk of virus transmission is clearly greater during these responses, particularly where oxygen administration is required [22]. Also elevating risk is the inability of staff to physically distance while responding to overdose.

An additional challenge for clinical staff was the scarcity of PPE in the early period of the outbreak, an issue that has also been reported internationally [23]. The fluid nature of information about COVID-19, and the often highly contested views in relation to transmission risks that have emerged throughout the pandemic [24], has also presented challenges in implementing procedural change.

Protocols evolved over time as community risk increased, evidence of risk exposures became available, and response strategies were trialled and assessed. For example, resuscitation protocols developed rapidly from requiring PPE for staff performing BVM resuscitation, to cessation of BVM altogether once the aerosolization risk of virus transmission was more clearly established. Requirements on the use of PPE continued to progress as understanding of the virus and transmission risks advanced, with differences between the MSIC and MSIR reflecting the substantive difference in levels of community transmission in each jurisdiction over time.

Procedural changes often needed to be implemented quickly, with decisions being made daily (sometimes hourly), based on the best available health advice. Communicating these changes, along with information about the virus and reducing transmission risk, has been crucial, as many clients may not regularly engage with other health services.

There were several challenges from a client perspective. Client feedback on procedural change, sought in the aftercare area, was incorporated as an important element of adaptation, reflecting one of the fundamental principles (client-centred) of drug treatment provision in Australia more broadly [25].

Firstly, reducing capacity in the registration area meant increased numbers of clients queuing outside. This increased client visibility, in turn increasing the potential for law enforcement intervention. Increased liaison between the SIFs and local police occurred during this time to facilitate greater understanding and ensure that clients could access the service without impediment. In an attempt to reduce unnecessary law enforcement engagement, and to adhere to public health guidelines, staff monitored the entrance to ensure clients were safely distancing.

Second, reducing time spent in the aftercare area meant clients were missing opportunities for incidental social interaction. MSIC re-instituted their consumer advisory group (CAG) meetings at the CAG’s request. This was to ensure some level of connectedness among clients, while also managing distancing requirements. Aftercare area restrictions also meant reduced opportunity for client-staff interactions regarding referrals and physical and mental health assessments. Staff were encouraged to be particularly vigilant and proactive in determining client needs.

The final challenge relates directly to virus transmission. As at the time of writing, there have been two confirmed COVID-19 cases among MSIR clients (none at MSIC and none among staff). Provision of COVID-19 testing onsite allowed for rapid detection and response. All procedures (described in Table 1) implemented at the MSIR meant that neither case necessitated closure of the service nor quarantine for staff, given none were considered in close contact. Monitoring of client movement throughout the centre was pivotal in contact tracing and facilitating further testing among clients where necessary.

Services continue to be monitored and procedures revised as government directives evolve. To the credit of both the MSIC and the MSIR staff, and their clients, these changes have been implemented while in a constant state of flux.

## Discussion

This paper shows the adaptability of Australian SIFs in responding to the evolving COVID-19 pandemic. Both services implemented significant changes to operating procedures to minimise transmission risks for clients and staff, as well as ensure continued provision of a safer supervised injecting space. Changes were implemented immediately following the introduction of national government restrictions in March, and in the absence of clear guidance on what these restrictions meant for local harm reduction services.

Such a proactive response was critical to continued service provision at both sites in a way that is consistent with government COVID-19 and public health guidelines. As with any health service, remaining open during this time poses potential risks of transmission for clients and staff. In the case of SIFs, the harm of the potential loss of life from injecting in unsafe/unsupervised environments and the loss of ancillary supports for people who inject drugs means ongoing service provision is important.

Physical distancing measures are likely to continue to be required to mitigate the risks of virus transmission [1]. Changes to overdose management may also need to be incorporated into standard operating procedures for the foreseeable future.

Important lessons have arisen from the experience of both services during this time. Firstly, one of the key features enabling the MSIC and MSIR to remain open during the pandemic, and to respond to risk appropriately, is that they operate within a clinical model. As such, they had access to the requisite medical supplies (e.g. PPE), albeit with some difficulty at times, enabling them to adjust protocols to continue operations, and to safely and effectively respond to overdose while minimising transmission risks.

Arguably there are also limitations to operating within the clinical model, including the limited capacity to provide outreach support for clients who may have been further isolated during the pandemic. Both services worked closely with local outreach teams in an attempt to address this gap in service delivery, with outreach teams extending their services to cover a greater number of clients across a larger geographic area.

Second, changes to service entry procedures and the use of PPE among staff appeared to create barriers between staff and clients, highlighting the importance of these relationships. Given the discrimination SIF clients often face in many health settings, it matters how clients are welcomed into these services, as well as the nature of staff–client interactions that occur while they are in the service.

Third, the utility of onsite COVID-19 testing at the MSIR was highlighted, with temperature screening providing additional benefits for clients such as identifying untreated septicaemia. The MSIR is likely to continue with these measures beyond the duration of the pandemic.

Finally, the reduction in capacity in the aftercare area of both services impacted clients negatively, through reduced social interaction. This has highlighted the role these services play, beyond safe spaces to inject, in providing important opportunities for social connectedness between clients.

It has been a worrying time for many frontline AOD services in Australia as they navigate uncertain conditions with respect to the pandemic, and brace for potentially unprecedented demand for drug treatment [1]. This may well exacerbate the already existing unmet treatment demand [26] during a particularly challenging time for many Australians.

The continued operation of the MSIC and MSIR during this time has been critical for people who inject drugs in Sydney and Melbourne, and the public health benefits for these groups profound. Preventing substantial loss of life and improving health and wellbeing among these groups has clearly been demonstrated by these services both in Australia and internationally [3, 6]. People who use drugs are often isolated (economically, socially and in their ability to access health services) under normal circumstances, and the pandemic has undoubtedly highlighted this isolation in many different ways.

It remains to be seen what longer term impacts COVID-19 and the resulting restrictions will have on illicit drug markets internationally. Any significant changes will have implications for drug use and related harms among people who use drugs [27]. While there are early reports from European countries that some drug use (e.g. cocaine and MDMA use) is declining due to restrictions on the night time economy, there are reports from other countries of substitution of heroin with other substances (including buprenorphine and amphetamines), and increasing reports of amphetamine use in Northern European countries [28]. There are also reports of increased attempts to access OAT in parts of Europe [28]. Indicators in Australia suggest a decline in frequency of injecting among some consumers in the context of declining access to methamphetamine and heroin [29], with other consumers reporting increases in alcohol and cannabis use [30].

Harm reduction services across North America have been greatly reduced, and some closed to minimise the risk of virus transmission [13, 15, 16]. In addition, opioid overdose deaths have steadily increased during the pandemic, particularly in Canada where they have reportedly increased by 40% after declining in the previous year [13]. Access to supervised consumption services in Canada has been greatly reduced due to restrictions, and often long wait times outside these services [16]. Without access to these services, the likelihood of people injecting alone increases, which in turn increases the risk of fatal overdose [13]. The impact of the pandemic on substance use patterns, onsite drug overdoses and the numbers of clients attending Australian SIFs will be the subject of future analyses.

Some of the future challenges for drug using populations are likely to be broader than drug-related harms, and future work assessing these impacts, including the ways in which these populations were engaged (or not) throughout the pandemic, is required. Further economic hardship may be inevitable, particularly as Australia moves into a recession and the current level of economic support provided to these populations is reduced [31]. Likewise, the mental health impacts present a significant challenge, with access to mental health services difficult for these groups [32].

In addition, social interaction has been greatly reduced across the world for these client groups, with many drop-in centres across Europe forced to close during the pandemic [14]. Supervised injecting facilities and DCRs worldwide play an important role in client advocacy, particularly in relation to access and equity. This role is likely to gain increasing importance as the longer term impacts of the pandemic emerge.

## Conclusion

The global COVID-19 pandemic has irrevocably altered the way we all live, and health services have been required to adapt to ever-changing government regulations. The MSIC and MSIR implemented substantial changes to the way they provide services including reducing numbers of clients within the service at any one time, adapting overdose protocols to ensure they could respond as safely as possible, and vastly increasing their capacity to facilitate access to temporary accommodation and OAT services. Both the MSIC and MSIR provide critical services for people who inject drugs and are important conduits to accessing other health and social services. Their continued operation during the pandemic has been essential. If anything is to be learnt from a global health emergency such as COVID-19, it is that we need to provide health care that is readily accessible and responsive to *all* community members, particularly the most vulnerable in our population.

## Data Availability

Data on COVID-19 cases in New South Wales and Victoria were downloaded from a publicly available website www.covid19data.com.au.
